# Impact of Multidirectional Transverse Calf Muscle Loading on Calf Muscle Force in Young Adults

**DOI:** 10.3389/fphys.2018.01148

**Published:** 2018-08-17

**Authors:** Tobias Siebert, Manuel Eb, David S. Ryan, James M. Wakeling, Norman Stutzig

**Affiliations:** ^1^Department of Motion and Exercise Science, University of Stuttgart, Stuttgart, Germany; ^2^Department of Biomedical Physiology and Kinesiology, Simon Fraser University, Vancouver, BC, Canada

**Keywords:** transverse load, muscle compression, human gastrocnemius, muscle contraction dynamics, compression garments, muscle pressure

## Abstract

It has been demonstrated that unidirectional transversal muscle loading induced by a plunger influences muscle shape and reduces muscle force. The interaction between muscle and transversal forces may depend on specific neuromuscular properties that change during a lifetime. Compression garments, applying forces from all directions in the transverse plane, are widely used in sports for example to improve performance. Differences in the loading direction (unidirectional vs. multidirectional) may have an impact on force generating capacity of muscle and, thus, on muscle performance. The aim of this study was to examine the effect of multidirectional transversal loads, using a sling looped around the calf, on the isometric force during plantarflexions. Young male adults (25.7 ± 1.5 years, *n* = 15) were placed in a prone position in a calf press apparatus. The posterior tibial nerve was stimulated to obtain the maximal double-twitch force of the calf muscles with (59.4 and 108.4 N) and without multidirectional transverse load. Compared to the unloaded condition, the rate of force development (*RFD*) was reduced by 5.0 ± 8.1% (*p* = 0.048) and 6.9 ± 10.7% (*p* = 0.008) for the 59.4 and 108.4 N load, respectively. No significant reduction (3.2 ± 4.8%, *p* = 0.141) in maximum muscle force (*F_m_*) was found for the lower load (59.4 N), but application of the higher load (108.4 N) resulted in a significant reduction of *F_m_* by 4.8 ± 7.0% (*p* = 0.008). Mean pressures induced in this study (14.3 and 26.3 mm Hg corresponding to the 59.4 and 108.4 N loads, respectively) are within the pressure range reported for compression garments. Taking the results of the present study into account, a reduction in maximum muscle force would be expected for compression garments with pressures ≥26.3 mm Hg. However, it should be noted that the loading condition (sling vs. compression garment) differs and that compression garments may influence other mechanisms contributing to force generation. For example, wearing compression garments may enhance sport performance by enhanced proprioception and reduced muscle oscillation. Thus, superposition of several effects should be considered when analyzing the impact of compression garments on more complex sport performance.

## Introduction

**FIGURE 1:**
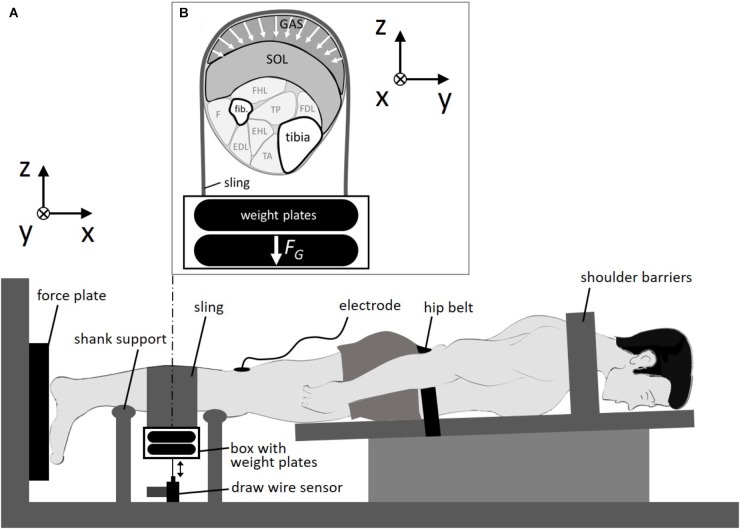
Experimental setup. **(A)** Subject was fixed in a prone position. The sling was looped around the calf and loaded by weight plates. **(B)** Cross section of the right calf. Calf muscles were compressed by the sling in multiple directions in the transversal plane due to gravitational forces (*F_G_*) of the weight plates. GAS: *M. gastrocnemius*; SOL, *M. soleus*; fib., fibula; FHL, *M. flexor hallucis longus*; FDL, M. flexor *digitorum longus*; TP, *M. tibialis posterior*; F, *M. fibularis*; EDL, *M. extensor digitorum longus*; EHL, *M. extensor hallucis longus*; TA, *M. tibialis anterior*; x, line of action/longitudinal direction; y-z plane, transversal plane.

Neuromuscular performance depends on the properties of the neuromuscular system, which change during the lifespan, as well as on the interaction of the neuromuscular system with external forces from the environment. The majority of knowledge about skeletal muscle force generation is based on experiments on muscle fiber preparations (Gordon et al., 1966; Edman, 1988; Ranatunga et al., 2007) and isolated muscles (Hill, 1938; Katz, 1939; Scott et al., 1996; Siebert et al., 2015) that are freed from the neighboring tissue. In this isolated situation, the muscle or muscle fiber can deform freely without any external forces (except for the gravitational force). This is in contrast to the *in vivo* situation where muscles are surrounded by other muscles, connective tissue, and bones. These neighboring tissues may transfer forces to the muscle, modifying muscle architecture (Wick et al., 2018) and the force generation in the longitudinal (in the direction of the line of action, **Figure 1**, x-axis) direction. Examination of the interaction of muscles with external forces is important for a better understanding of movement generation and control. Myofascial force transmission via fascial connections between neighboring muscles has been shown to influence longitudinal muscle force (Carvalhais et al., 2013; Bernabei et al., 2015; Yucesoy et al., 2015). Furthermore, transverse compressive forces (perpendicular to the line of action) may be transferred between muscles during contraction thereby potentially influencing the longitudinal muscle force (Reinhardt et al., 2016).

External forces from outside the body can also act on muscles in addition to forces transferred from neighboring structures. Kinesio taping influences passive muscle shape and architecture (Pamuk and Yucesoy, 2015) and changes at least the initial conditions of a contraction. Muscle compression induced by transverse forces commonly occurs in daily life (for instance when wearing compression garments and during sitting) and the impact of compression garments on human performance is of great interest. Strength and power performance after fatigue seem to recover at a faster rate with the use of compression garments, for instance by increasing venous return and removal of metabolites (Hill et al., 2013), limitation of oedema (Partsch, 2012), and increased oxygen delivery to the tissue (Bringard et al., 2006). However, the effect of compression garments on acute sporting performance and muscle force is currently under discussion (Heneghan et al., 2012).

Studies on isolated rat muscles (Siebert et al., 2014a,b, 2016) revealed that muscle compression in unilateral transverse direction (**Figure 1**, z-axis) induced by a plunger reduced muscle work and force (up to 15%) performed in the longitudinal direction as well as the rate of force development (*RFD*) (up to 35%). Unidirectional transverse muscle loading may also be experienced during sitting. In contrast, compression garments (e.g., compression pants) induce external forces that act on the muscle from all directions in the transverse plane (multidirectional transverse loading, **Figure 1**, y-z plane). These different loading situations (unidirectional vs. multidirectional loading) lead to different passive muscle deformations and may subsequently lead to different contraction dynamics.

Experimental studies examining the impact of multidirectional transverse loading on muscle force and deformation are rare. Wakeling et al. (2013) reported reduced muscle thickness and fascicle pennation during cyclical isotonic plantarflexions with elastic compression bandages around the calf muscles. Azizi et al. (2017) constrained the muscle by a rigid tube and observed reduced muscle shortening and work. However, both studies didn’t measure directly the amount of multidirectional transverse loading and its effect on muscle force.

Muscle deformations during contraction are strongly influenced by muscle architecture as well as active and passive muscle properties, which change dramatically during aging (Siebert et al., 2017). Thus, mechanical interaction between muscle and transversal loads might be age dependent. Here we induced a specific multidirectional transverse loading and measured its impact on muscle force and *RFD* in young adults. This was achieved by a sling that was looped around the human calf and loaded with two different weights (59.4 and 108.4 N) during electrically induced plantarflexions. It was hypothesized that multidirectional transversal muscle loading leads to a reduction in longitudinal muscle force.

## Materials and Methods

### Subjects

Fifteen male young adults (height: 179 ± 6 cm; weight: 77.6 ± 7.2 kg; BMI: 24.1 ± 1.2; age: 25.7 ± 1.5 years) participated in this study. All subjects were informed about the risks of the experiments and gave their written consent. The study protocol was approved by the ethical committee of the university hospital of Tuebingen and conducted according to the latest declaration of Helsinki.

### Measurement of Muscle Force and Lifting Height of the Load

We measured the force generated by the calf muscles during a double-twitch stimulation applied to the posterior tibial nerve. The double-twitch force was used as it is highly reproducible (Stutzig and Siebert, 2016) and commonly used to examine contraction dynamics in human experiments (Stutzig and Siebert, 2015a,b). The cathode was fixed in the popliteal fossa as close as possible to the posterior tibial nerve and the anode (5 × 10 cm) was fixed on the thigh approximately 2 cm proximal to the patella. A trigger box (DG2A, Digitimer, Herfordshire, United Kingdom) and a high current stimulator (DS7AH Digitimer, Herfordshire, United Kingdom) generated the electrical paired stimuli (pulse interval 10 ms, pulse duration: 1 ms). The maximal stimulation intensity was assessed using a ramp protocol. Starting at 10 mA, the current was increased by 10 mA every 10 s until the double-twitch force did not further increase. A 3D force plate (Type 9260 AA3, Kistler Instrumente AG, Winterthur, Switzerland, sample rate: 1,000 Hz) was used to measure the double-twitch force during stimulation. The vertical displacement of the load was recorded using a draw wire sensor (SX50, WayCon, Taufkirchen, Germany, sample rate: 1,000 Hz), which was connected to the load (**Figure 1**). The lift height *Δh* of the load was calculated as the difference between its maximal height during contraction and its initial height in the passive state when the passive muscle was compressed. Work performed to lift the load *W_lift_* was calculated by multiplying *Δh* and the gravitational force (load 1:59.4 N, load 2:108.4 N).

### Experimental Protocol

At first the subjects performed a standardized warm-up consisting of 5 min of running on a treadmill (with 12 km/h), 3 sets of 10 calf raises (bilateral), and 10 repetitive calf jumps (bilateral). Afterward, the subjects were instructed to lie prone in a calf press apparatus with the right foot attached to a 3D force plate (**Figure 1**). The left foot was placed and secured beside the force plate. The stimulation electrodes were attached on the skin and the subject was immobilized in this position (full extended knee, ankle angle at 90°) with shoulder barriers and with a hip belt. Subjects then performed a further warm-up consisting of 10 submaximal (increasing from about 30 to 90% maximum voluntary contraction) isometric plantarflexions. First stimulation was performed about 5 min after the warm up.

The experiments started with five paired stimuli, applied to the posterior tibial nerve every 10 s at rest for the pretest baseline. Then, a leather sling (width: 15 cm, length: 75 cm) was looped around the calf at its most prominent bulge (**Figure 1**) covering the gastrocnemius muscle belly for the most part (about 70%). The longitudinal distance of the proximal border of the sling to the popliteal fossa (where the cathode was fixed) was about 7 cm. An aluminum box was hung on both ends of the sling for storage of the metal weights (5 or 10 kg). The draw wire sensor was connected via a hook to the bottom of the box. Due to the pulling force (3 N) of the draw wire sensor, and the deadweight of the aluminum box (0.5 kg) and sling (0.25 kg), loading of the box with 5 and 10 kg resulted in transverse loads of 59.4 and 108.4 N, respectively. We performed two series of loaded experiments (load 1:59.4 N, load 2:108.4 N) each consisting of 5 paired stimuli. In between the two series a rest time of 1 min was set without transverse muscle loading. Finally, five double-twitches every 10 s were performed without transverse muscle loading (post-test) to assess possible conditional changes during the experiment.

### Data Analysis

A moving average filter (window length: 11 samples) was used for smoothing the force and kinematic data. The resultant force was calculated from the measured 3D force components for each double-twitch and used to quantify the maximal double-twitch force (*F_m_*) and the *RFD* using custom-made Matlab scripts (MATLAB R2013a, The MathWorks, Inc., Natick, MA, United States). *RFD* was calculated as the force difference between 0.1 and 0.9 *F_m_* divided by the corresponding required time. The mean and standard deviation of a stimulation series (e.g., 5 doublets at rest) was assessed and used for further statistical analyses.

### Statistical Analyses

Data are presented as mean ± standard deviation. No indication of deviation from normal distribution was found using the Shapiro–Wilk Test. As lifting height of the load was measured for the loaded conditions only, a *t*-test for dependent samples was performed to test for differences between 59.4 and 108.4 N load. One-way repeated measures ANOVA (rANOVA) were conducted for the force parameters *(F_m_, RFD*) to test for differences between conditions (pretest, 59.4 N load, 108.4 N load, and post-test). The level of significance was set at *p* < 0.05. Significant main effects or interactions of the rANOVA were probed using Bonferroni *post-hoc* tests. Effect size was determined using partial eta squared (η_p_^2^*)* and classified as follows: low η_p_^2^ = 0.01, medium η_p_^2^= 0.06, and large η_p_^2^ = 0.14 (Cohen, 1988).

## Results

**FIGURE 2:**
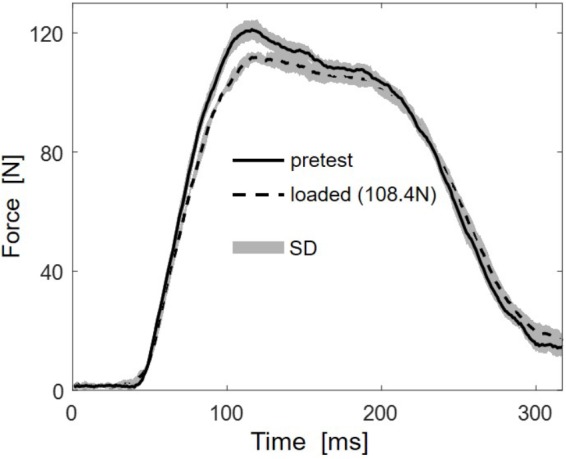
Representative (subject 10) mean force-time traces of five stimulations for the highest load (108.4 N, dashed line) and the five unloaded reference contractions (pretest, solid line). Standard deviations (SD) are shown as gray areas.

Multidirectional transverse muscle loading affected the force generation during plantarflexion. **Figure 2** shows the mean force-time curve of 5 stimulations for the higher load (108.4 N) and the mean of 5 unloaded reference contractions. The force-time curves indicate reduced *RFD* and reduced maximal force.

**FIGURE 3:**
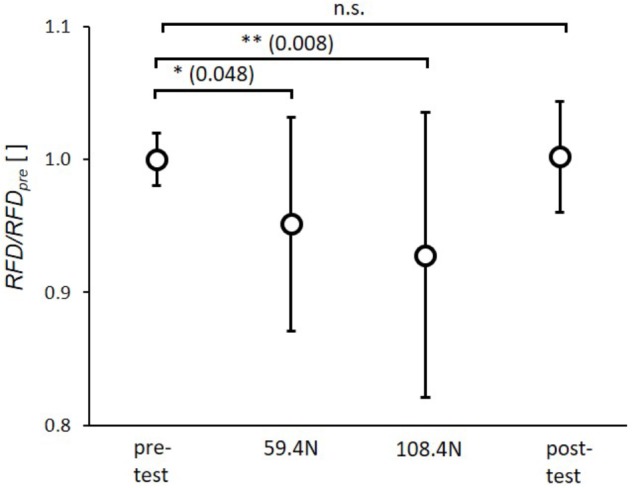
Mean (circles) and standard deviation of rate of force development (*RFD*) for pretest, loading conditions (59.4 and 108.4 N) and post-test. For each subject *RFD* values of single twitches were normalized by mean subject specific rate of force development of pretest (*RFD_pre_*). ^∗^*p* < 0.05; ^∗∗^*p* < 0.01; ^∗∗∗^*p* < 0.001.

The statistical analysis showed significant effects of transverse loading for *RFD* (*p* < 0.001, η_p_^2^= 0.33). The *post hoc* tests revealed a significant reduction of *RFD* by 5.0 ± 8.1% and 6.9 ± 10.7% between pretest and 59.4 N load and between pretest and 108.4 N load (**Figure 3**), respectively. There were no significant differences between pre- and post-test as well as between 59.4 and 108.4 N loading.

**FIGURE 4:**
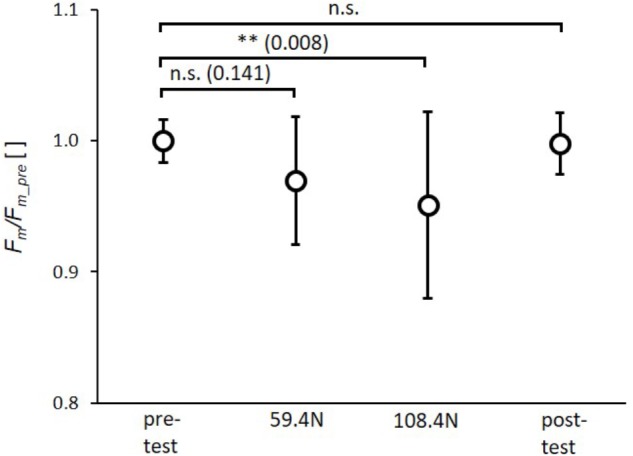
Mean (circles) and standard deviation of maximal force (*F_m_*) for pretest, loading conditions (59.4 and 108.4 N) and post-test. For each subject *F_m_* values of single twitches were normalized by mean subject specific maximal force of pretest (*F_m_pre_*). ^∗^*p* < 0.05; ^∗∗^*p* < 0.01; ^∗∗∗^*p* < 0.001.

The rAnova revealed significant effects of muscle loading for *F_m_*(*p* = 0.003, η_p_^2^= 0.28). The *post-hoc* test revealed a significant reduction in *F_m_* by 4.8 ± 7.0% (*p* = 0.008) for the higher load (108.4 N). The reduction in *F_m_* of 3.2 ± 4.8% induced by the lower load (59.4 N) was not significant (**Figure 4**). Furthermore, there were no significant differences between the 59.4 and 108.4 N load (*p* = 0.141) as well as between pre- and post-test.

Due to problems with the data acquisition of the lift height for one subject, only data of 14 subjects have been analyzed. During plantarflexion contractions, the load was lifted by 4.6 ± 1.2 mm and by 5.6 ± 2.0 mm for the lower (59.4 N) and the higher (108.4 N) load, respectively. A *t*-test for dependent samples showed no significant differences between the lift heights (*p* = 0.064). The work performed to lift the load was higher (*p* < 0.001) for the 108.4 N load (616 ± 213 mJ) compared to the 59.4 N load (273 ± 70 mJ).

## Discussion

The aim of the present study was to examine the effect of multidirectional transverse muscle loading on contraction dynamics. Here we used a sling looped around the calf to apply multidirectional transverse muscle loading to the human calf muscles. Our experimental results demonstrate that muscle force and *RFD* were decreased by 4.7% and 6.9% in the loaded (108.4 N) condition, respectively, compared to the unloaded condition. In general, our observations are consistent with experiments applying unidirectional transversal forces on isolated rat muscles (Siebert et al., 2014a, 2016) and reporting reduced muscle forces, too. As transverse muscle loading (unidirectional and multidirectional) increases intramuscular pressure, a general reduction in muscle force can be explained by this effect (Siebert et al., 2018). However, application of transversal forces (0.64–2.6 N) by a plunger to the rat *M. gastrocnemius medialis* (GM) resulted in a more pronounced reduction of *F_m_* (4.8–12.8%) and *RFD* (20–35%) (Siebert et al., 2014b). This might be due to differences in the normalized amount of loading, the loading condition (multidirectional vs. transversal), or the observed specimen (isolated rat GM vs. human calf muscles).

Normalization of transversal forces (0.64–2.6 N) used in rat experiments by the maximum isometric GM muscle force (*F_im_* = 11.2 N; Siebert et al., 2014a) resulted in values of 6–23% of *F_im_*. Applying a similar normalization for the transversal forces in this study (*F_im_*= 4,500 N for human calf muscles at knee and ankle angles of 180° and 90°; Haxton, 1944; Stutzig and Siebert, 2015a) would yield much lower normalized muscle loading of 1–2% of *F_im_* for loads of 59.4 and 108.4 N, respectively. As the reduction in muscle force and *RFD* increase with increasing loads (Siebert et al., 2014b, 2016), lower normalized muscle loading might partially explain lower force and *RFD* reduction in the present study.

Furthermore, differences in the loading condition might influence force-producing mechanisms. In the present study, applied forces act in multiple directions within the transverse (z-y) plane (**Figure 1**) compared to the unidirectional action of forces in z-direction in the plunger experiments. This impacts the muscle shape and architecture during passive muscle loading and changes at least the initial conditions of a contraction. Moreover, uni- and multidirectional transverse muscle loading may result in different deformations of the myofilament grid which might have impact on force generation capacity of cross bridges (Siebert et al., 2018).

Comparison of results of unidirectional and multidirectional transverse muscle loading is even more difficult as different specimens (isolated rat GM vs. human calf muscles) have been observed. For a single muscle, the reduction in muscle force can be mainly described by a simple model approach using a lever to convert transverse force and length change (i.e., lifting height) into longitudinal force and length (Siebert et al., 2014a, 2018). In the present study, multidirectional transverse forces acted on the gastrocnemius muscle (**Figure 1B**) and it is unclear if these forces were transmitted to the underlying *M. soleus* (SOL) as the depth of compression was not ascertained in this study. Therefore, specific contributions of GAS and SOL to calf deformations (and thus lifting height of the load) and reduction in longitudinal muscle force are unknown. In principle, transmission of transversal forces between neighboring muscles is possible (Reinhardt et al., 2016) allowing the different muscles to work together within a muscle package. Additionally, it is likely that calf muscle deformation and lifting height of the load was influenced by deformations of the smaller and deeper-lying FHL, TP, and FDL (**Figure 1B**). However, more precise examinations of 3D muscle architectures of calf muscles and their deformations during contraction are required to gain a better understanding of 3D interactions of synergistic muscles with each other as well as with external transverse forces.

There are only two studies that have applied multidirectional forces in the transverse plane to the muscle, but with deviating experimental approaches. Azizi et al. (2017) limited the radial expansion of isolated palmaris longus muscles (*n* = 4) of leopard frogs. The authors constructed small rigid plastic tubes, fitted to the diameter of the muscle, and placed them around the muscle. Small reductions of about 5% in maximum isometric force were reported, but were not significant (from 1.66 ± 0.36 N to 1.58 ± 0.30 N). Furthermore, the amount of muscle shortening and work output was reduced significantly during isotonic contractions against 25% *F_im_* in the constraint condition. Wakeling et al. (2013) applied elastic compression bandages to the human lower leg. This external compression reduced muscle thickness, fascicle pennation and force generating capacity of fascicles, but the isotonic experimental design controlled the plantarflexions to have similar ankle torque and angular velocity. Summarizing, it can be stated, that restriction of radial muscle expansion by application of multidirectional transverse forces (e.g., by rigid tubes, elastic bandages, or slings) affects muscle deformation, muscle architecture and mechanical performance, which potentially influences muscle force and shortening as well as the ability of the muscle to do work in the longitudinal direction.

Measured mean forces of contractions without transverse muscle loading evoked by electrical stimulation are 165 ± 30 N. Considering a gearing of 2.8 (ratio of moment arms of the foot and the calcaneus about the angle joint; Arndt et al., 1998), this corresponds to Achilles tendon forces of 462 ± 84 N. Reported maximum values for Achilles tendon forces are in the range of 489–661 N during cycling at work loads between 88 and 265 W (Gregor et al., 1987) and in the range of 1,320–1,490 N during walking at speeds of 1.1 and 1.8 m/s (Finni et al., 1998). Thus, electrically evoked forces are in the lower range of forces produced during voluntary efforts.

### Restriction of Muscle Deformation by Compression Garments and Increased ECM Stiffness

Restriction of muscle deformation by multidirectional transverse forces may be relevant when wearing compression garments as well as when examining the effects of increased stiffness of the collagenous extracellular matrix (ECM) on muscle performance. For example, compression garments are increasingly used by elite and recreational athletes, as they are thought to enhance performance. However, the results concerning increased acute sport performance are controversial (Beliard et al., 2015; Donath and Faude, 2016). Kraemer et al. (1996) found no effect on maximal force or power of the highest vertical jump, but did show enhanced mean force and power over 10 jumps. Mixed results for jumping performance and no effect on sprinting were reported by MacRae et al. (2011) regarding the use of compression garments, while Born et al. (2013) calculated small positive effect sizes for vertical jumping and sprint performance when compression garments were worn. Wearing compression garments had no impact on running (Ali et al., 2007) and cycling performance (Scanlan et al., 2008). Pressures applied at the calf by 12 different compression garments out of 24 studies analyzed in a review article (Beliard et al., 2015) are in the range of 8–39 mm Hg (corresponding to 0.10–0.52 N/cm^2^) with a mean value of 19.9 mm Hg (0.26 N/cm^2^). These pressures are within the range of mean pressures (transverse load divided by contact area between muscle and sling) of 0.19 ± 0.02 and 0.35 ± 0.03 N/cm^2^ induced in the present study with loads of 59.4 and 108.4 N, respectively. Taking these results into account, pressure induced by compression garments might be in a range where no effect on muscle force can be expected (**Figure 4**, 59.4 N load corresponding to ∼0.19 N/cm^2^ ∼14.3 mm Hg). Since we found a significant decrease in muscle force of 4.8% for the higher transverse load (**Figure 4**, load 108.4 N), a decrease in muscle force might be expected for compression garments that induce a higher pressure (≥0.35 N/cm^2^∼26.3 mm Hg). However, results concerning the impact of compression garments on acute sport performance are controversial, as stated above. Regarding this, it should be noted that compression garments may influence other mechanisms contributing to force generation (which might be negligible in the current study) and counteract a potential reduction in muscle force induced by muscle compression. Wearing compression garments may enhance sport performance through enhanced proprioception (Perlau et al., 1995; Bernhardt and Anderson, 2005), increased muscle blood flow (Broatch et al., 2017), and reduced muscle oscillation (Sperlich et al., 2013) during repetitive exercise, which might reduce muscle pain and fatigue (Kraemer et al., 1998; Doan et al., 2003). Furthermore, measurements of complex voluntary performances (as in cycling, sprinting, jumping) have higher mean variations compared to experiments with electrical muscle stimulation on immobilized subjects. Thus, it might be possible that such small differences as an expected reduction in force of 5% induced by muscle compression cannot be resolved during complex voluntary movements exhibiting variations in force >5%.

In addition to compression garments, slings, or elastic bandages, increased stiffness of the ECM restricts muscle deformation. The mechanical properties of the ECM change with age, exercise, or illness (Kjaer et al., 2006; Gao et al., 2008; Lieber and Ward, 2013). Alnaqeeb et al. (1984) reported increased muscle stiffness with age that was closely correlated with the increase in endomysium, perimysium, and with total muscle collagen content. Moreover, ECM production is enhanced after chronic eccentric muscle loading (Heinemeier et al., 2007; Franchi et al., 2017). A stiffer ECM was found in children with spastic cerebral palsy (Smith et al., 2011) and as a consequence of skeletal muscle fibrosis (Lieber and Ward, 2013). Simulations of muscle contractions with increasing ECM stiffness restricted the muscle’s ability to expand radially, which in turn compromises muscle shortening and performance (Azizi et al., 2017). Moreover, muscle architecture (e.g., pennation angle) and muscle properties change with age (Alnaqeeb and Goldspink, 1987; Binzoni et al., 2001; Siebert et al., 2017), and depend on the specific type of training (Franchi et al., 2014, 2017) resulting in age and training dependent muscle shape, size, and deformation during contraction. Better understanding of mechanical interaction between contractile tissue and transverse forces (e.g., induced by compression garments or slings) in young healthy adults may help to reveal how an age related deviation (e.g., in ECM stiffness) can compromise performance in older people or in response to neuromuscular pathologies.

## Author Contributions

TS, NS, JW, and DR conceived and designed the experiments. ME performed the experiments. TS, NS, and ME analyzed the data. TS and DR prepared the figures. All authors interpreted the results, edited, revised, and drafted the manuscript, and approved the final version of manuscript.

## Conflict of Interest Statement

The authors declare that the research was conducted in the absence of any commercial or financial relationships that could be construed as a potential conflict of interest.
